# Intrafacet Spacer Placement as a Mobility-Sparing Bailout Option in Atlantoaxial Fusion Construct Salvage

**DOI:** 10.31486/toj.23.0080

**Published:** 2024

**Authors:** Tyler Scullen, James Milburn, Mansour Mathkour, R. Shane Tubbs, James Kalyvas

**Affiliations:** ^1^ Department of Neurological Surgery, Ochsner Clinic Foundation, New Orleans, LA; ^2^ Department of Neurological Surgery, Tulane University School of Medicine, New Orleans, LA; ^3^ Department of Radiology, Ochsner Clinic Foundation, New Orleans, LA; ^4^ The University of Queensland Medical School, Ochsner Clinical School, New Orleans, LA

**Keywords:** *Arthrodesis*, *atlanto-axial joint*, *neck pain*, *reoperation*

## Abstract

**Background:** Salvage revisions of atlantoaxial (AA) joint complex posterior segmental instrumented fusion constructs require careful individualized planning to prevent occipital extension. In this case report, we describe the use of bilateral intrafacet spacer placement as a mobility-sparing bailout option for the revision surgery.

**Case Report:** A 64-year-old male with a history of diffuse idiopathic skeletal hyperostosis, extremely limited baseline cervical mobility, and prior AA posterior segmental instrumented fusion presented with increasing pain at his 6-month follow-up. Imaging showed fusion and hardware failures and dynamic instability. To prevent occipitocervical fixation, AA intra-articular fusion via a DTRAX spinal system (Providence Medical Technology, Inc) was used as an adjunct to a navigated C1 lateral mass and C2 pars screw posterior segmental instrumented fusion construct. The patient had an uneventful postoperative course and was discharged with resolution of symptoms. Three-month postoperative follow-up confirmed persistent resolution of symptoms and absence of complaints, along with successful arthrodesis on imaging.

**Conclusion:** AA posterior segmental instrumented fusion revision is technically challenging, particularly when partial preservation of craniovertebral junction mobility is required. Bilateral intra-articular cages may be used as an adjunct to hardware revision in construct salvage when sturdy arthrodesis is desired without occipital extension and may represent a major potential strength of intra-articular cages.

## INTRODUCTION

Instrumented arthrodesis has become a mainstay of contemporary spinal surgery and a focus for optimization and advancement.^1-10^ Treatment around the atlantoaxial (AA) joint complex of the craniovertebral junction (CVJ) is particularly challenging given regional pathology, biomechanics, and anatomy.^11^ Posterior segmental instrumented fusion, historically consisting of bilateral posterolateral screw and rod constructs, demands advanced consideration in the setting of revision procedures because successful arthrodesis can prevent occipitocervical fixation and the associated impacts on quality of life.^10-14^

We present a case of successful revision of failed AA posterior segmental instrumented fusion constructs using adjunct bilateral AA intrafacet spacers.

## CASE REPORT

**Figure 1.**
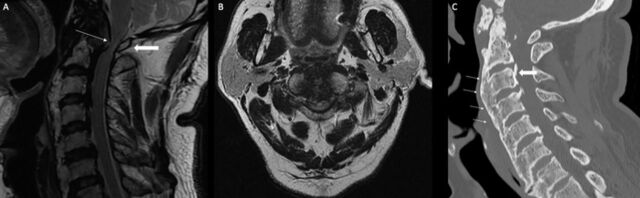
Noncontrast T2-weighted (A) sagittal and (B) axial magnetic resonance imaging of the cervical spine obtained on initial presentation shows significant canal stenosis (block arrow) at the craniovertebral junction secondary to significant ventral soft tissue compression and tectorial membrane buckling (arrow). (C) Noncontrast sagittal computed tomography of the cervical spine shows diffuse idiopathic skeletal hyperostosis with continuous anterior (arrows) and segmental posterior (block arrow) ossified longitudinal ligaments extending into the anterior atlanto-occipital and tectorial membranes.

**Figure 2.**
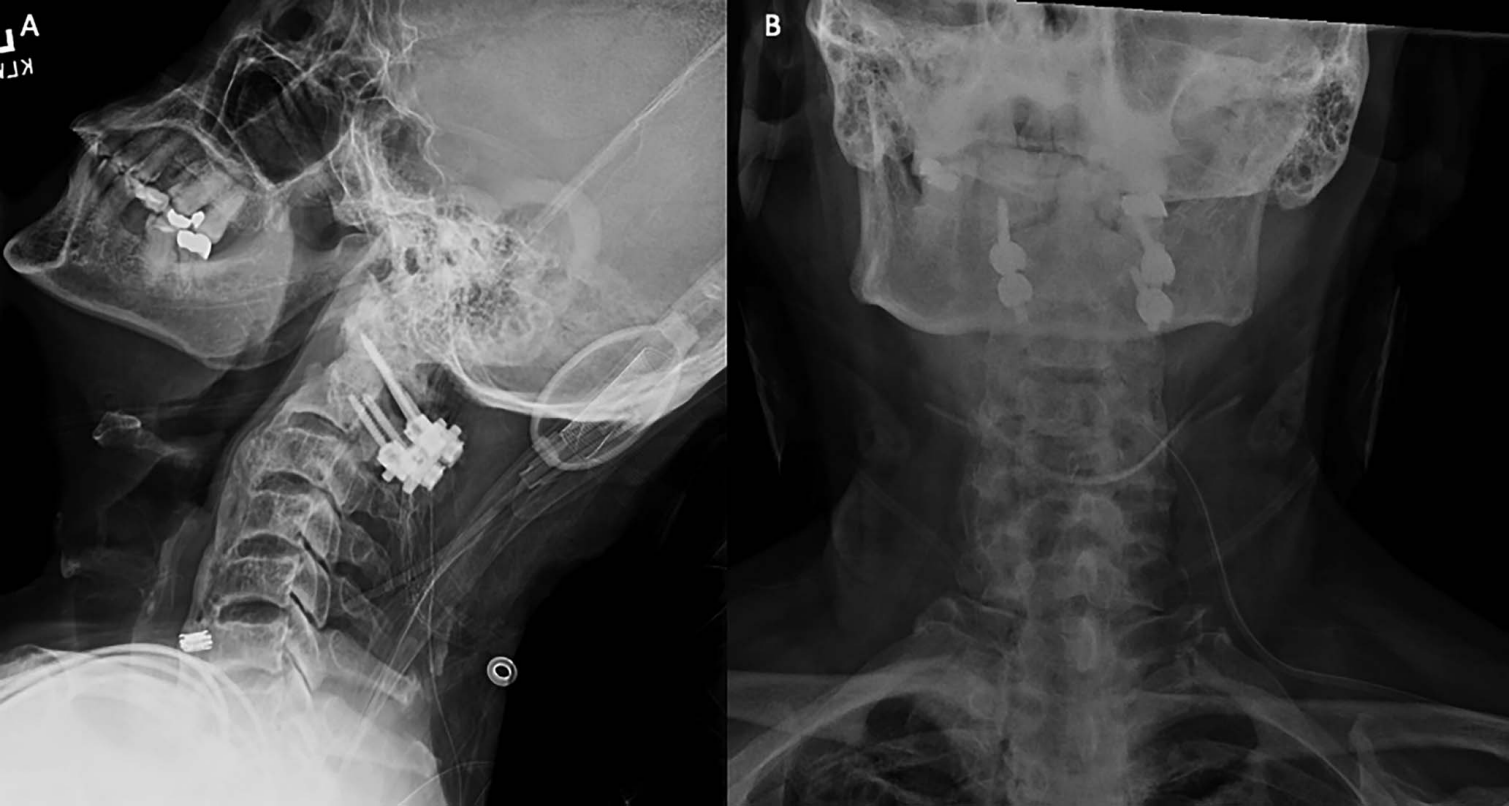
(A) Lateral and (B) anteroposterior plain films of the cervical spine show the initial construct of bilateral lateral mass and pars screws across C1 and C2.

A 64-year-old male with a history of diffuse idiopathic skeletal hyperostosis presented after a mechanical fall with worsening myelopathy. Cervical imaging demonstrated nontraumatic CVJ stenosis secondary to an anterior AA membrane pannus, continuous ossification of the anterior longitudinal ligament extending into the distal anterior atlanto-occipital membrane, and segmental ossification of the posterior longitudinal ligament with minimal involvement of the tectorial membrane (Figure 1). Given worsening symptoms, the patient was treated by C1 laminectomy and AA posterior segmental instrumented fusion with bilateral C1 lateral mass and C2 pars screws (Figure 2).

**Figure 3.**
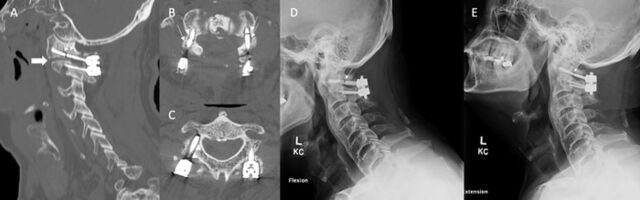
Noncontrast computed tomography (A) sagittal view of the craniovertebral junction at the plane of the left facet demonstrates a fractured C1 lateral mass screw (small arrows) and a C2 pars articularis screw with the tip in the region of the C2 transverse foramen. Failed arthrodesis is noted at the atlantoaxial articulation (block arrow), which was consistent contralaterally. Axial sequences at the (B) C1 and (C) C2 levels show bilateral fractured lateral mass screws (arrows) and pseudoarthrosis of the right pars screw (white arrowheads on medial aspect). Lateral (D) flexion and (E) extension dynamic plain films of the cervical spine show movement of the fractured lateral mass screws (arrows).

Following an uncomplicated discharge with resolution of myelopathy, the patient reported progressive CVJ pain at his 6-month follow-up. Dynamic imaging to evaluate arthrodesis showed fusion and hardware failures, with bilateral lateral mass screw fractures, pseudoarthrosis of the right pars screw, and movement of the C1 screw fracture segments (Figure 3).

The patient underwent hardware exploration and hardware removal via an open midline approach with the distal lateral mass screw segments retained within C1. The AA joint was exposed bilaterally using a modified Harms approach.^12^ The dorsal rami and venous plexuses of the C2 spinal nerves were found to be adherent and were transected. Intraoperative guidance was registered via surface matching of the C2 posterior elements to preoperative imaging using Brainlab Spine Navigation (Brainlab AG) to cannulate screw tract trajectories that avoided retained hardware.

Attention was turned to the AA facets, which were sharply incised, and the intra-articular space was prepared using a DTRAX spinal system (Providence Medical Technology, Inc). Bilateral 4-mm DTRAX Cervical Cage-B intrafacet implants (Providence Medical Technology, Inc) were packed with demineralized bone matrix (Johnson & Johnson) and inserted under fluoroscopy. The surrounding surfaces were decorticated, and the construct was completed with rods adjoined to freehand bilateral lateral mass screws (3.5 × 30 mm, Johnson & Johnson) and pars screws (3.5 × 18 mm, Johnson & Johnson) placed within the prepared tracts.

**Figure 4.**
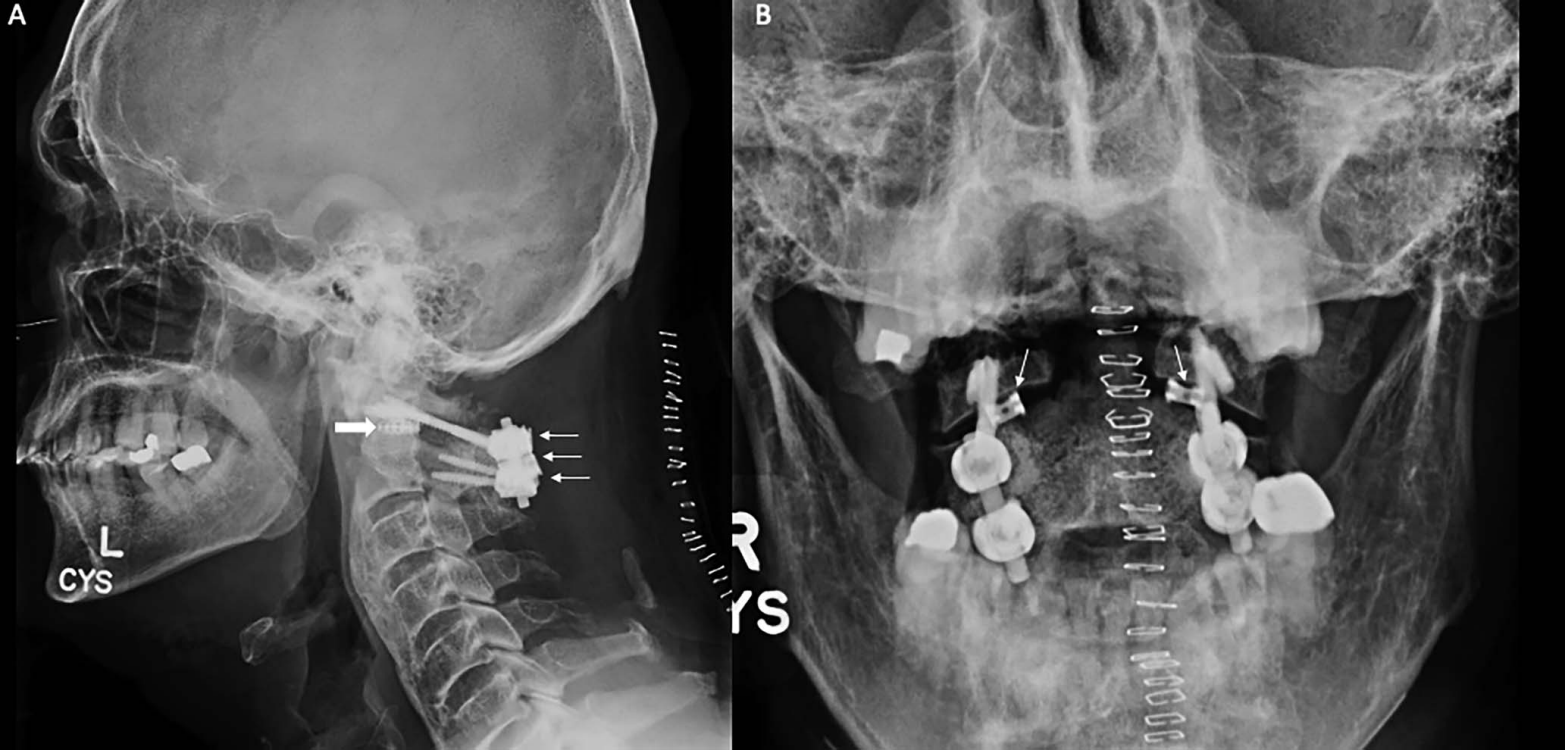
Postoperative plain films of the craniovertebral junction. (A) Lateral view shows successful placement of revision hardware across C1 and C2 with bilateral lateral mass and pars screws (arrows), along with intrafacet implants at the lateral atlantoaxial joints (block arrow). (B) Open mouth anteroposterior odontoid view shows standard posterolateral construct combined with bilateral intrafacet C1-C2 spacer placement (arrows).

**Figure 5.**
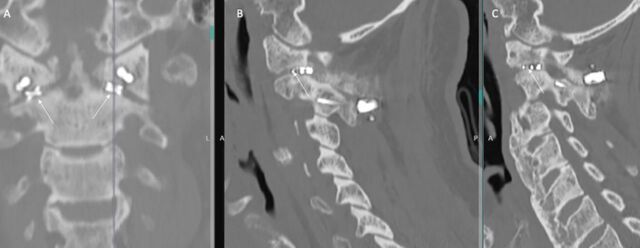
Noncontrast cervical spine computed tomography (A) coronal and (B, C) sagittal sequences centered on the (B) right and (C) left atlantoaxial joints show solid arthrodesis through the intrafacet constructs (arrows).

Final imaging (Figure 4) and transcortical motor evoked potentials were satisfactory. The patient had an uneventful postoperative course and was discharged with symptom resolution. Three-month follow-up confirmed persistent resolution of symptoms, absence of complaints, and successful arthrodesis (Figure 5).

## DISCUSSION

AA joint complex arthrodesis failure significantly affects quality of life and is reported with variable frequency in adults.^1-12,15-41^ While most literature describes high rates of success in the setting of C1 lateral mass and C2 pars screw posterior segmental instrumented fusion constructs,^32-38,40-42^ revision rates remain high, at upwards of 20%.^33-37,43^ In the United States between 1993 and 2014, primary C1-C2 arthrodesis surgeries increased 111% annually, while between 2006-2014, revision surgeries at the same level decreased by only 6% per year, with a resultant 57% proportional increase in hospital cost secondary to revisions.^10^ Recent years have also seen multiple reports of alternative techniques aimed at optimizing AA posterior segmental instrumented fusion constructs,^38,42^ despite a reported 95% to 100% arthrodesis rate in most series.^32-40,42-44^

The authors speculate that the high rate of revision surgeries required in ostensibly successful fusions may be associated with ambiguity in assessment parameters and terminology.^32-40,42-45^ Indications for revision are often cited as “neck pain” or patient-reported failure rather than failure of constructs.^32,33,35-37,43^ Many authors define stability on dynamic plain films as a surrogate for arthrodesis success, assuming perceived gross stability and bony fusion to be synonymous.^33-40,42-45^ Regardless, a 2022 report that used dynamic stability to report fusion outcomes described fusion failure in up to 20% of patients following Harms technique AA posterior segmental instrumented fusion, which is likely to be a more accurate estimate of successful arthrodesis compared to studies boasting 100% success rates.^41^

Therapeutic arthrodesis manipulates bone healing responses following cortical breach or articular destruction, with hardware accelerating ossification and maintaining stability during proliferation of inflammatory mesenchymal derivatives during the acute phase.^27,28^ The added rigidity is critical in revision procedures, where fibrous scar tissue has already blanketed the area, creating a microenvironment that does not promote fusion and distorts normal anatomy.^29^ Intrafacet cage systems have been described to supplement both posterior and anterior constructs, with commercial systems approved as an adjunct to posterior segmental instrumented fusion constructs for subaxial cervical spondylosis.^15-26^ Additional anecdotal application has been described with good short-term outcomes during occipitocervical revision and naïve AA posterior segmental instrumented fusion constructs in both pediatric and adult populations.^13,19^ Rationales for concurrent intrafacet constructs involve observed segmental biomechanical rigidity comparable to subaxial interbody fusion, with continued joint distraction and ligamentous tension.^19,25,26^

Increased rigidity along previously mobile joints also raises force magnitudes placed on adjacent articulations, increasing susceptibility to adjacent segment disease, which has been reported secondary to intrafacet spacer placement.^25^ The AA joint complex is known to compensate for subaxial spine spondylosis, risking adjacent segment disease in both AA and subaxial posterior segmental instrumented fusion constructs.^12,42,45^ Additionally, long-segment AA constructs spanning the subaxial cervical spine increase stress on the highly mobile proximal complex, particularly at C1 lateral mass screws in scenarios where decompression mandates disruption of the posterior tension band.^12,42,45^ In such cases, consideration of occipitocervical extension to increase fixation points and dissipate strain has been advocated.^12,42,45^ The authors speculate that our patient developed his presenting disease from such processes, the extended lever of the autofused cervical spine inducing continuous CVJ strain, ongoing inflammation, retro-odontoid pannus formation, and a loss of joint integrity. The baseline lack of mobility in this case increased our desire to fortify the AA construct rigidity, preserving the atlanto-occipital joint to prevent depriving the patient of virtually all CVJ mobility. As such, adjunctive intrafacet spacers were considered as a possible solution to increase segmental rigidity in a difficult anatomic region under increased stress with iatrogenically limited potential fixation points. Furthermore, while the AA joint complex is easily located in naïve patients, it was anatomically distorted in our patient, with a difficult postsurgical haptic and visual environment.

AA joint complex revisions characteristically have fewer potential fixation points compared to first-time procedures in the setting of bone resection and resorption, often necessitating extension.^13,14^ Hardware fracture increases complexity, limiting potential screw trajectories and accentuating the restraints of regional neurovascular anatomy, with increased vertebral artery injury risk being associated with alternative screw trajectories.^30^ Vertebral artery integrity is also a major consideration in primary fusions for AA joint complex trauma, which account for most primary and iatrogenic spinal vascular injuries.^12,42,45^ The supplemented use of intrafacet spacers, especially in revision or trauma surgeries with displaced or resected fixation options and increased vertebral artery injury risk, could provide a potentially quality of life–improving alternative, avoiding occipitocervical posterior segmental instrumented fusion constructs or less efficacious fallback constructs.^31,32^

We suggest that major indications of intrafacet fusion include AA posterior segmental instrumented fusion revisions; AA joint complex trauma cases with a high risk of vertebral artery injury; and AA cervicothoracic constructs where increased proximal fixation and the risk of vertebral artery injury can preclude the need for occipitocervical extension, risking additional morbidity.^13,19,22^ While the literature describing the use of intrafacet spacers in first-time AA posterior segmental instrumented fusion reports good fusion rates and outcomes,^13,18,26^ evidence of successful fusion in revision cases remains limited, and our conclusion is anecdotal despite the impressive lateral AA arthrodesis observed in our patient at 3 months. We note that the resolution of pain in our case, a significant source of morbidity for the patient, could have been at least partially influenced by the bilateral C2 neurectomies. However, that is not to say that the spacers did not contribute to the observed symptom improvement, because increased intervertebral foraminal area and reduction of radiculopathy symptoms have been described in the subaxial spine and in nonclinical models.^23-25^ The observed symptom improvement was noted on a short-term follow-up period of 3 months, and continued monitoring is necessary to confirm durability of treatment.

## CONCLUSION

Revision of the AA joint complex segment is technically and strategically challenging, particularly in scenarios with an increased need to avoid occipitocervical extension and preserve a degree of CVJ mobility. We describe the successful novel application of bilateral intra-articular cages as an adjunct to AA posterior segmental instrumented fusion construct salvage in a patient with hardware failure and a known history of diffuse idiopathic skeletal hyperostosis. We suggest that revision procedures are a major potential strength of intra-articular arthrodesis, with the likelihood of further application to trauma cases with risk of injury to the vertebral artery and to proximal AA joint complex fixation of long-segment constructs.

## Notes

*This article meets the Accreditation Council for Graduate Medical Education and the American Board of Medical Specialties Maintenance of Certification competencies for Patient Care and Medical Knowledge.*

## References

[1] Goel A, Shah A. Facetal distraction as treatment for single- and multilevel cervical spondylotic radiculopathy and myelopathy: a preliminary report. J Neurosurg Spine. 2011;14(6):689-696. doi: 10.3171/2011.2.SPINE10601 21417697

[2] Conrad BP, Cordista AG, Horodyski M, Rechtine GR. Biomechanical evaluation of the pullout strength of cervical screws. J Spinal Disord Tech. 2005;18(6):506-510. doi: 10.1097/01.bsd.0000140196.99995.65 16306839

[3] Tjardes T, Shafizadeh S, Rixen D, Image-guided spine surgery: state of the art and future directions. Eur Spine J. 2010;19(1):25-45. doi: 10.1007/s00586-009-1091-9 19763640 PMC2899744

[4] Dong J, Lu M, Lu T, Artificial disc and vertebra system: a novel motion preservation device for cervical spinal disease after vertebral corpectomy. Clinics (Sao Paulo). 2015;70(7):493-499. doi: 10.6061/clinics/2015(07)06 26222819 PMC4496753

[5] Carvalho AD, Figueiredo J, Schroeder GD, Vaccaro AR, Rodrigues-Pinto R. Odontoid fractures: a critical review of current management and future directions. Clin Spine Surg. 2019;32(8):313-323. doi: 10.1097/BSD.0000000000000872 31464693

[6] Patel PD, Canseco JA, Houlihan N, Gabay A, Grasso G, Vaccaro AR. Overview of minimally invasive spine surgery. World Neurosurg. 2020;142:43-56. doi: 10.1016/j.wneu.2020.06.043 32544619

[7] Fiani B, Nanney JM, Villait A, Sekhon M, Doan T. Investigational research: timeline, trials, and future directions of spinal disc arthroplasty. Cureus. 2021;13(7):e16739. doi: 10.7759/cureus.16739 34513367 PMC8405360

[8] Sun Y, Yang F, Ma HN, Occipitocervical revision surgery using the bicortical screw and plate system for failed craniovertebral junction stabilization. Orthop Surg. 2022;14(2):238-245. doi: 10.1111/os.13086 34904372 PMC8867430

[9] Xu HT, Zheng S, Dong RP, Yu T, Zhao JW. Combined 3-dimensional printing model and 3-dimensional fluoroscopic navigation to assist C2 pedicle screw insertion: a case report. Medicine (Baltimore). 2020;99(43):e21838. doi: 10.1097/MD.0000000000021838 33120726 PMC7581057

[10] Hendow CJ, Beschloss A, Cazzulino A, Change in rates of primary atlantoaxial spinal fusion surgeries in the United States (1993-2015). J Neurosurg Spine. 2020;32(6):900-906. doi: 10.3171/2019.11.SPINE19551 31978892

[11] Fiore AJ, Haid RW, Rodts GE, Atlantal lateral mass screws for posterior spinal reconstruction: technical note and case series. Neurosurg Focus. 2002;12(1):E5. doi: 10.3171/foc.2002.12.1.6 16212332

[12] Buchmann N, Schweizer C, Kirschke JS, C1-C2 posterior screw fixation in atlantoaxial fractures revisited: technical update based on 127 cases. Eur Spine J. 2020;29(5):1036-1042. doi: 10.1007/s00586-019-06244-2 31823086

[13] Duan W, Chou D, Jiang B, Posterior revision surgery using an intraarticular distraction technique with cage grafting to treat atlantoaxial dislocation associated with basilar invagination. J Neurosurg Spine. 2019;31(4):525-533. doi: 10.3171/2019.4.SPINE1921 31277061

[14] Goel A, Dhar A, Shah A, Rai S, Bakale N, Vaja T. Revision for failed craniovertebral junction stabilization: a report of 30 treated cases. World Neurosurg. 2019;127:e856-e863. doi: 10.1016/j.wneu.2019.03.286 30954741

[15] McCormack BM, Bundoc RC, Ver MR, Ignacio JM, Berven SH, Eyster EF. Percutaneous posterior cervical fusion with the DTRAX Facet System for single-level radiculopathy: results in 60 patients. J Neurosurg Spine. 2013;18(3):245-254. doi: 10.3171/2012.12.SPINE12477 23330952

[16] Cofano F, Sciarrone GJ, Pecoraro MF, Cervical interfacet spacers to promote indirect decompression and enhance fusion in degenerative spine: a review. World Neurosurg. 2019;126:447-452. doi: 10.1016/j.wneu.2019.03.114 30904796

[17] Yazdanshenas H, Osias E, Hwang R, Park DY, Lord E, Shamie AN. Retrospective evaluation of cervical fusion with DTRAX (R) cervical cage. J Craniovertebr Junction Spine. 2022;13(1):48-54. doi: 10.4103/jcvjs.jcvjs_150_21 35386243 PMC8978854

[18] Sommer F, Kirnaz S, Goldberg JL, Safety and feasibility of DTRAX Cervical Cages in the atlantoaxial joint for C1/2 stabilization. Oper Neurosurg (Hagerstown). 2022;22(5):322-327. doi: 10.1227/ons.0000000000000139 35315806

[19] Kramer S, Albana MF, Ferraro JB, Shah RV. Minimally invasive posterior cervical fusion with facet cages to augment high-risk anterior cervical arthrodesis: a case series. Global Spine J. 2020;10(2 Suppl):56S-60S. doi: 10.1177/2192568220911031 32528806 PMC7263338

[20] Cheng L, McCormack B, Eyster EF. Posterior cervical fusion utilizing cages placed bilaterally in the facets for the treatment of the upper cervical adjacent segment disease in the elderly. J Clin Neurosci. 2019;63:149-154. doi: 10.1016/j.jocn.2019.01.018 30732988

[21] Voronov LI, Siemionow KB, Havey RM, Carandang G, Patwardhan AG. Biomechanical evaluation of DTRAX(®) posterior cervical cage stabilization with and without lateral mass fixation. Med Devices (Auckl). 2016;9:285-290. doi: 10.2147/MDER.S111031 27601934 PMC5003555

[22] Voronov LI, Siemionow KB, Havey RM, Carandang G, Phillips FM, Patwardhan AG. Bilateral posterior cervical cages provide biomechanical stability: assessment of stand-alone and supplemental fixation for anterior cervical discectomy and fusion. Med Devices (Auckl). 2016;9:223-230. doi: 10.2147/MDER.S109588 27471414 PMC4948702

[23] Siemionow K, Janusz P, Phillips FM, Clinical and radiographic results of indirect decompression and posterior cervical fusion for single-level cervical radiculopathy using an expandable implant with 2-Year follow-up. J Neurol Surg A Cent Eur Neurosurg. 2016;77(6):482-488. doi: 10.1055/s-0036-1584210 27276119

[24] Siemionow K, Janusz P, Glowka P. Cervical cages placed bilaterally in the facet joints from a posterior approach significantly increase foraminal area. Eur Spine J. 2016;25(7):2279-2285. doi: 10.1007/s00586-016-4430-7 26869077

[25] Siemionow K, Monsef JB, Janusz P. Preliminary analysis of adjacent segment degeneration in patients treated with posterior cervical cages: 2-year follow-up. World Neurosurg. 2016;89:730.e1-730.e7307. doi: 10.1016/j.wneu.2016.01.053 26836696

[26] Li S, Ni B, Xie N, Biomechanical evaluation of an atlantoaxial lateral mass fusion cage with C1-C2 pedicle fixation. Spine (Phila Pa 1976). 2010;35(14):E624-E632. doi: 10.1097/BRS.0b013e3181cf412b 20505567

[27] Silva JC, Carvalho MS, Udangawa RN, Extracellular matrix decorated polycaprolactone scaffolds for improved mesenchymal stem/stromal cell osteogenesis towards a patient-tailored bone tissue engineering approach. J Biomed Mater Res B Appl Biomater. 2020;108(5):2153-2166. doi: 10.1002/jbm.b.34554 31916699

[28] Hsia AW, Emami AJ, Tarke FD, Osteophytes and fracture calluses share developmental milestones and are diminished by unloading. J Orthop Res. 2018;36(2):699-710. doi: 10.1002/jor.23779 29058776 PMC5877458

[29] Kaliya-Perumal AK, Ingham PW. Musculoskeletal regeneration: a zebrafish perspective. Biochimie. 2022;196:171-181. doi: 10.1016/j.biochi.2021.10.014 34715269

[30] Pragash V, Douraiswami B, Subramani S. Axis vertebral dimensions for safe screw placement: a CT normative data analysis. J Clin Orthop Trauma. 2020;11(5):871-875. doi: 10.1016/j.jcot.2020.06.026 32879573 PMC7452170

[31] Cadena G, Duong HT, Liu JJ, Kim KD. Atlantoaxial fixation using C1 posterior arch screws: feasibility study, morphometric data, and biomechanical analysis. J Neurosurg Spine. 2018;30(3):314-322. doi: 10.3171/2018.8.SPINE18160 30554179

[32] Ni B, Zhao W, Guo Q, Comparison of outcomes between C1-C2 screw-hook fixation and C1-C2 screw-rod fixation for treating reducible atlantoaxial dislocation. Spine (Phila Pa 1976). 2017;42(20):1587-1593. doi: 10.1097/BRS.0000000000002152 28296813

[33] Lall R, Patel NJ, Resnick DK. A review of complications associated with craniocervical fusion surgery. Neurosurgery. 2010;67(5):1396-1403. doi: 10.1227/NEU.0b013e3181f1ec73 20871441

[34] Elliott RE, Morsi A, Frempong-Boadu A, Smith ML. Is allograft sufficient for posterior atlantoaxial instrumented fusions with screw and rod constructs? A structured review of literature. World Neurosurg. 2012;78(3-4):326-338. doi: 10.1016/j.wneu.2011.12.083 22381276

[35] Coyne TJ, Fehlings MG, Wallace MC, Bernstein M, Tator CH. C1-C2 posterior cervical fusion: long-term evaluation of results and efficacy. Neurosurgery. 1995;37(4):688-693. doi: 10.1227/00006123-199510000-00012 8559297

[36] Gautschi OP, Payer M, Corniola MV, Smoll NR, Schaller K, Tessitore E. Clinically relevant complications related to posterior atlanto-axial fixation in atlanto-axial instability and their management. Clin Neurol Neurosurg. 2014;123:131-135. doi: 10.1016/j.clineuro.2014.05.020 25012025

[37] Shi L, Shen K, Deng R, Novel unilateral C1 double screw and ipsilateral C2 pedicle screw placement combined with contralateral laminar screw-rod fixation for atlantoaxial instability. Eur Spine J. 2019;28(2):362-369. doi: 10.1007/s00586-018-5853-0 30539243

[38] Larsen AMG, Grannan BL, Koffie RM, Coumans JV. Atlantoaxial fusion using C1 sublaminar cables and C2 translaminar screws. Oper Neurosurg (Hagerstown). 2018;14(6):647-653. doi: 10.1093/ons/opx164 28962019

[39] Dogan S, Gundogdu EB, Taşkapılıoğlu MÖ, Karaoglu A. Rod migration to the thoracic subarachnoid space after C_1-2_ instrumentation: a case report and literature review. Orthop Surg. 2017;9(1):129-132. doi: 10.1111/os.12317 28371499 PMC6584166

[40] Lee SH, Kim ES, Sung JK, Park YM, Eoh W. Clinical and radiological comparison of treatment of atlantoaxial instability by posterior C1-C2 transarticular screw fixation or C1 lateral mass-C2 pedicle screw fixation. J Clin Neurosci. 2010;17(7):886-892. doi: 10.1016/j.jocn.2009.10.008 20399666

[41] Su ML, Liu ZH, Tu PH, Huang YC. Dynamic cervical flexion/extension atlantodental interval and functional outcome of the Harms technique for posterior C1/2 fixation: a retrospective analysis of 16 atlantoaxial subluxation cases in a tertiary medical center. Neurochirurgie. 2022;68(2):168-174. doi: 10.1016/j.neuchi.2021.10.003 34774580

[42] Koffie RM, Larsen AMG, Grannan BL, Novel technique for C1-2 interlaminar arthrodesis utilizing a modified Sonntag loop-suture graft with posterior C1-2 fixation. Neurospine. 2020;17(3):659-665. doi: 10.14245/ns.1938344.172 32054143 PMC7538353

[43] Robinson LC, Anderson RCE, Brockmeyer DL, Torok MR, Hankinson TC; Pediatric Craniocervical Society. Comparison of fusion rates based on graft material following occipitocervical and atlantoaxial arthrodesis in adults and children. Oper Neurosurg (Hagerstown). 2018;15(5):530-537. doi: 10.1093/ons/opy013 29554356 PMC6186910

[44] Chen Q, Chen J, Chen F, Lu X, Ni B, Guo Q. Biomechanics of the effect of subaxial cervical spine degeneration on atlantoaxial complex in idiopathic retro-odontoid pseudotumor development. Clin Neurol Neurosurg. 2020 Oct 16;106314. doi: 10.1016/j.clineuro.2020.106314 34756393

[45] Ghaith AK, Yolcu YU, Alvi MA, Rate and characteristics of vertebral artery injury following C1-C2 posterior cervical fusion: a systematic review and meta-analysis. World Neurosurg. 2021;148:118-126. doi: 10.1016/j.wneu.2020.12.165 33516865

